# Inhibition of Inflammatory and Proliferative Responses of Human Keratinocytes Exposed to the Sesquiterpene Lactones Dehydrocostuslactone and Costunolide

**DOI:** 10.1371/journal.pone.0107904

**Published:** 2014-09-16

**Authors:** Claudia Scarponi, Elena Butturini, Rosanna Sestito, Stefania Madonna, Andrea Cavani, Sofia Mariotto, Cristina Albanesi

**Affiliations:** 1 Experimental Immunology Laboratory, IDI-IRCCS, Rome, Italy; 2 Department of Life and Reproduction Sciences, University of Verona, Verona, Italy; Carl-Gustav Carus Technical University-Dresden, Germany

## Abstract

The imbalance of the intracellular redox state and, in particular, of the glutathione (GSH)/GSH disulfide couple homeostasis, is involved in the pathogenesis of a number of diseases. In many skin diseases, including psoriasis, oxidative stress plays an important role, as demonstrated by the observation that treatments leading to increase of the local levels of oxidant species ameliorate the disease. Recently, dehydrocostuslactone (DCE) and costunolide (CS), two terpenes naturally occurring in many plants, have been found to exert various anti-inflammatory and pro-apoptotic effects on different human cell types. These compounds decrease the level of the intracellular GSH by direct interaction with it, and, therefore, can alter cellular redox state. DCE and CS can trigger S-glutathionylation of various substrates, including the transcription factor STAT3 and JAK1/2 proteins. In the present study, we investigated on the potential role of DCE and CS in regulating inflammatory and proliferative responses of human keratinocytes to cytokines. We demonstrated that DCE and CS decreased intracellular GSH levels in human keratinocytes, as well as inhibited STAT3 and STAT1 phosphorylation and activation triggered by IL-22 or IFN-γ, respectively. Consequently, DCE and CS decreased the IL-22- and IFN-γ-induced expression of inflammatory and regulatory genes in keratinocytes, including CCL2, CXCL10, ICAM-1 and SOCS3. DCE and CS also inhibited proliferation and cell-cycle progression-related gene expression, as well as they promoted cell cycle arrest and apoptosis. In parallel, DCE and CS activated the anti-inflammatory EGFR and ERK1/2 molecules in keratinocytes, and, thus, wound healing in an *in vitro* injury model. In light of our findings, we can hypothesize that the employment of DCE and CS in psoriasis could efficiently counteract the pro-inflammatory effects of IFN-γ and IL-22 on keratinocytes, revert the apoptosis-resistant phenotype, as well as inhibit hyperproliferation in the psoriatic epidermis.

## Introduction

Pathogenic mechanisms leading to the expression of T-cell mediated skin disorders, such as psoriasis, are mostly driven by T lymphocytes belonging to the functional subsets T helper (Th)1, Th17, Th22, and T cytotoxic 1 lymphocytes. In inflamed skin lesions, these T cells produce massive amounts of cytokines (i. e. IFN-γ, TNF-α, IL-17 and IL-22), which in synergy induce potent inflammatory responses in resident skin cells, primarily keratinocytes [1], [2], [3]. Upon exposure to T-cell cytokines, in particular IFN-γ, keratinocytes become a source of immune mediators, which in turn creates a favourable milieu leading to a rich inflammatory infiltrate in the whole skin including the upper layers of the epidermis, and eventually to the aggravation and/or perpetuation of the skin disorder [4], [5]. In addition, specific cytokines, such as IL-22, can trigger regenerative and proliferative programs in keratinocytes as well as induce antimicrobial peptides, and hence can be centrally involved in the pathogenesis of skin diseases characterized by epidermal hyperproliferation, including psoriasis [6], [7], [8]. Consistently with the activating lymphokine, keratinocytes show dynamic molecular cascades that ultimately induce the activation of transcription factors and downstream genes controlling inflammation and/or cell growth/differentiation. For instance, the transcription factors signal transducer and activator of transcription (STAT)3 and STAT1 are aberrantly activated in the epidermis of psoriatic lesions by IL-22 and IFN-γ, respectively, and control the production of CCL2, CXCL10 and CXCL8 chemokines, the expression of β-defensins HBD-2 and HBD-3, as well as of genes involved in cell-cycle progression (i. e. cyclin D1, PCNA, and p-RB) in keratinocytes [8], [9]. However, in parallel, epidermal growth factor receptor/mitogen activated protein (EGFR/MAP) kinase and AKT molecular pathways can be up-regulated in skin lesions to counteract inflammatory and apoptotic responses in keratinocytes, respectively. EGFR/MAP kinase activation has been shown to down-regulate the expression of a cluster of chemokines whose expression is triggered by TNF-α and IFN-γ, including CCL2, CXCL8, whereas AKT controls the anti-apoptotic NF-κB and the pro-apoptotic BAD cascades [10], [11]. In psoriatic keratinocytes, the increased AKT activation is responsible for the enhanced resistance to apoptosis typical of the disease. Therefore, any treatment counteracting the hyper-activation of STAT3 and STAT1 or controlling EGFR/MAP kinase and AKT pathways in keratinocytes can be reasonably considered as a strategy to treat skin immune-mediated disorders.

The intracellular redox state is a critical mediator of many metabolic, signaling and transcriptional processes in cells, and an adequate balance between oxidizing and reducing conditions is essential for normal function and cell survival [12], [13]. The glutathione/glutathione disulfide (GSH/GSSG) couple constitutes the major redox buffer in cytosol and plays a pivotal role in the maintenance of cellular reducing environment and defence against oxidative stress. The disturbance in the [GSH]/[GSSG] homeostasis is involved in the pathogenesis of a number of diseases. GSH deficiency or a decrease in the [GSH]/[GSSG] ratio manifests itself largely through an increased susceptibility to oxidative stress, as observed in Parkinson's and Alzheimer's disease. Conversely, elevated GSH levels increase antioxidant capacity and resistance to oxidative stress, and this is observed in cancer cells [13], [14].

In many skin diseases, such as psoriasis, allergic contact dermatitis and atopic dermatitis, oxidative stress is severely increased both systemically and locally in skin lesions. Interestingly, a variety of effective treatments used for therapy of psoriasis relies on a boost of the oxidative stress itself [15], [16]. For instance, treatments with psoralen-UVA, fumaric acid or anthralin, leading to increase of the local levels of oxidant species, ameliorate psoriasis, possibly due to activation of anti-proliferative, pro-apoptotic pathways in pathogenetic T cells and resident skin cells [17], [18], [19]. Recently, dehydrocostuslactone (DCE) and costunolide (CS), two naturally occurring terpenes present in a number of plants, such as *Laurusnobilis L.*, *Magnolia sieboldii L.*, and *Saussureacostus L.*, have been found to exert various anti-inflammatory and pro-apoptotic effects on different human cell types [20], [21]. These compounds decrease the level of the intracellular GSH by direct interaction with it, and, therefore, can alter cellular redox state. DCE and CS show an inhibitory action on a number of signal transduction pathways, among which STAT3, whereas activate many MAP kinases, such as JNK, ERK2 and p38 [22], [23]. At molecular level, DCE and CS directly interact with GSH, thereby triggering S-glutathionylation of STAT3 and other substrates. This event leads to a reduced STAT3 tyrosine phosphorylation and activation in response to the inducing cytokine IL-6. Concomitantly to STAT3, the tyrosine Janus kinase (JAK)1, JAK2 and Tyk2 are also de-phosphorylated in presence of DCE and CS [22].

In the present study, we hypothesized that the mild oxidative stress induced by DCE and CS treatments regulate inflammatory, proliferative and apoptotic responses of keratinocytes to inflammatory cytokines or in basal conditions. We found that these two terpenes substantially inhibited STAT3 and STAT1 pathways in keratinocytes, whereas enhance EGFR and ERK1/2 activation. DCE and CS decreased the expression of genes involved in cell-cycle progression and proliferation, and, in parallel, induced apoptosis and mitosis-arrest of keratinocytes. Interestingly, DCE and CS promoted wound healing in an *in vitro* injury model by enhancing keratinocyte migratory capabilities.

## Material and Methods

### Chemicals

DCE and CS were purchased from PhytoLab GmbH & Co. (Vestenbergsgreuth, Germany). Dehydrocostunolide (HCS), a structural analogue of CS lacking only the α-β-unsaturated carbonyl group, was synthesized and kindly provided by Prof. G. Appendino (University of Piemonte Orientale, Novara, Italy).

### GSH content quantification

The intracellular GSH concentration was measured by endpoint spectrophotometric titration on a Jasco V/550 spectrophotometer (JASCO, Cremella, Italy) using the 5,5′-dithiobis(2-nitrobenzoic acid) (DTNB, Ellman's reagent), as previously described [22]. Briefly, treated and untreated cells were lysed by freezing and thawing in 100 mM sodium phosphate buffer (PBS) pH 7.5, containing 5 mM EDTA, and after centrifugation at 16,000 rpm for 10 minutes, total protein concentration was determined by using Bradford method. The supernatants were de-proteinized with 5% trichloroacetic acid. For GSH measurement, acidified clear supernatants were neutralized and buffered at pH 7.4 with 200 mM K_2_HPO_4_, pH 7.5. The reaction was then started by the addition of 60 µM DTNB, and increase in absorbance at 412 nm was measured until no variation in absorbance was evident. The amount of total GSH was determined by comparison with GSH standard curve.

### Keratinocyte cultures and treatments

Human keratinocytes were obtained from skin biopsies of healthy donors, as previously reported [24]. Skin biopsies were obtained after patient's informed written consent, with the approval of the IDI-IRCCS Local Ethics Committee (17/3/2009, Prot. N. 33/CE/2009; study: “Immunità innata ed adattativa nella psoriasi: nuovi target molecolari per la diagnosi e la terapia”), and in conformity with the Helsinki guidelines. Second- or third-passage keratinocytes were used in all experiments, with cells cultured in the serum-free medium KGM (Clonetics, San Diego, CA), for at least 3–5 days (at 60–80% confluence) before performing cytokine treatment. Cells were pretreated with DCE, CS and HCS (all at 12.5 µM) for 1 h, and then stimulated with 50 ng/ml human recombinant IL-22, 200 U/ml human recombinant IFN-γ or 50 ng/ml human recombinant TNF-α (all from R&D Systems, Minneapolis, MN) in keratinocyte basal medium (KBM-GOLD, Clonetics) for different time periods. Cytotoxicity of DCE, HCS and CS was tested by measuring the activity of lactate dehydrogenase (LDH) released from keratinocyte cultures, using Cytotoxicity Detection Kit Plus-LDH (Roche Diagnostics, Milan, Italy), following the manufacturer' instructions.

### Western blotting

Total proteins, cytosolic and nuclear extracts were prepared as previously reported [24], and subjected to SDS-PAGE. Western blotting filters were developed using the ECL-plus detection system (Amersham, Dubendorf, Switzerland) or the SuperSignal West Femto kit (Pierce, Rockford, IL). The Abs employed for the study were as follows: anti-phospho-STAT3 (Serine 727, Ser727), anti-STAT3 (C-20), anti-phospho-STAT1 (Tyrosine 701, Tyr701), anti-STAT1 (E-23), anti-phospho-ERK1/2 (E-4), anti-ERK1/2 (C16), anti-phospho-EGFR (Tyr1173), anti-EGFR (1005), anti-Akt (H-136), anti-PCNA (PC10), anti-cyclin D1 (DCS-6), anti-β-actin (C-11), all provided by Santa Cruz Biotechnology (Santa Cruz, CA). Anti-phospho-STAT3 (Tyr705), anti-acetyl-STAT3 (lysine 685, Lys685), anti-phospho-STAT1 (Ser727), anti-phospho-Akt (Ser473), and anti-phospho-Retinoblastoma (RB) (Ser795) were from Cell Signaling Technology (Denvers, MA). Filters were properly developed with anti-mouse or anti-rabbit Ig Abs conjugated to HRP.

### Transient transfection and luciferase assay

Cultured keratinocytes grown in six-well plates were transiently transfected with the STAT3-responsive plasmid pLucTKS3 (a generous gift of Prof. J. Turkson, University of Central Florida, Orlando, FL) or pGASLuc plasmid by using Lipofectin reagent (InVitrogen), according to the manufacturer's instructions. At 24 h post-transfection cells were pretreated with 12.5 µM of DCE, CS and HCS for 1 h and then stimulated with IL-22 or IFN-γ for 8 h. After cell lysis in an appropriate buffer (Promega Italia, Milan, Italy), *Firefly* luciferase activity was measured using Dual-Glo Luciferase Assay System (Promega). To normalize the transfection efficiency, pRL-null plasmid encoding the *Renilla* luciferase was included in each transfection. Luciferase activity was further normalized by total cellular protein content assayed using Bradford (Sigma-Aldrich, Milan, Italy).

### RNA isolation and real-time polymerase chain reaction (PCR)

Total RNA from treated keratinocytes was extracted using the TRIzol reagent (InVitrogen), mRNA was reverse-transcribed into cDNA and analyzed by real-time PCR. The expression of suppressor of cytokine signalling (SOCS)3, SOCS1, ICAM1, CXCL10, IRF1, CCL2, S100A7, IL-20, HBD-2, and HPRT1 mRNA was evaluated in the ABI Prism SDS 7000 PCR instrument (Applied Biosystems, Branchburg, NJ), using SYBR Green PCR reagents or Taqman PCR Master Mix. The forward and reverse primers employed for real-time PCR were as follows: for SOCS3, 5′-AAGGACGGAGACTTCGATTCG-3′ and 5′-AAACTTGCTGTGGGTGACCAT-3′; for SOCS1, 5′-TTTTTCGCCCTTAGCGTGA-3′ and 5′-AGCAGCTCGAAGAGGCAGTC-3′; for ICAM1, 5′-GCTTCGTGTCCTGTATGGCC-3′ and 5′-TTTCCCGGACAATCCCTCTC-3′; for CXCL10, 5′-TGGCATTCAAGGAGTACCTCTCT-3′ and 5′-CTGATGCAGGTACAGCGTACG-3′; for IRF1, 5′-AAGGCCAAGAGGAAGTCATGTG-3′ and 5′-CCATCAGAGAAGGTATCAGGGC-3′; for CCL-2, 5′-CACCAGCAGCAAGTGTCCC-3′ and 5′-CCATGGAATCCTGAACCCAC-3′. The sequences of the primers and internal probe for HBD-2 mRNA have been previously described [8]. Primers for S100A7, IL-20 and HPRT-1 were provided by Applied Biosystems (HS 00161488, HS 00218888 and HS 4333768 respectively). The levels of gene expression were determined by normalizing to HPRT-1 mRNA expression. The values obtained from triplicate experiments were averaged, and data are presented as means ± SD.

### Crystal violet assay

2×10^4^ cells were seeded in 96-well plates, and, the day after, starved in KBM. Culture stimulation with IL-22 was conducted either in the presence or absence of 12.5 µM DCE, CS or HCS. After 2 d of treatment, cells were stained with 0.5% crystal violet, whose incorporation was measured at 540 nm in an ELISA reader (model 3550 UV ELISA reader; Bio-Rad, Hercules, CA).

### Cell Cycle Analysis

To determine cell-cycle distribution analysis, cells were cultured in 6-well plates, and, at 60–80% confluence, treated with vehicle alone (0.1% DMSO) or 12.5 µM of DCE, CS and HCS for 16 h. After treatment, cells were collected by trypsinization, fixed in 70% ethanol, washed in PBS, resuspended in PBS containing 1 mg/mL RNase and 50 µg/mL propidium iodide (PI), incubated in the dark for 30 min at room temperature, and analyzed with a flow cytometer (Becton Dickinson, Mountain View, CA). The data were analyzed using Multicycle software (Phoenix Flow Systems, San Diego, CA).

### Apoptosis analysis

Human keratinocytes were cultured in 6-well plates and, at 60–80% confluence, treated with DCE, CS and HCS for 1 h, and then stimulated with 50 ng/ml human recombinant TNF-α for 48 h. Apoptosis of keratinocytes was evaluated using the Genzyme TACS Annexin V apoptosis detection kit (R&D Systems) or the Cell Death Detection ELISA Plus kit (Roche Diagnostics). Viable, necrotic and apoptotic cells were analyzed by flow cytometry with a FACScan equipped with Cell Quest software (Becton Dickinson). The amount of nucleosomes was detected and quantified in cell lysates using Cell Death Detection ELISA Plus kit (Roche diagnostics), as per the manufacturer' instructions.

### Scratch wound healing assay

Keratinocytes were cultured in 6-well plates and, at 100% confluence, were either incubated or not with 10 µg/ml mitomycin C (Sigma-Aldrich) for 2 h. Cell were then scratched with the tip of a p-200 pipette to create a uniform cell-free zone. Wounded monolayers were pretreated with 12.5 µM DCE, CS and HCS for 1 h, and then stimulated with human recombinant IL-22 (50 ng/ml) for 12 h or 18 h. Microscopy pictures were taken with a digital camera at the different time points. The residual gap between migrating keratinocytes was measured with a computer-assisted image analysis system (Axiovision; Zeiss, Oberkochen, Germany), and expressed as percentage of the initial scratched area.

### Densitometric analysis and statistical analysis

Immunoblots were subjected to densitometry using an imaging densitometer (GS-670; Bio-Rad). The significance of differences between densitometric values was determined by Wilcoxon's signed rank test (SigmaStat; Jandel, San Rafael, CA). This test was also used to calculate the significance of differences between keratinocytes treated with DCE, HCS or CS and untreated cells in luciferase assays, real-time PCR analysis, crystal violet and DNA fragmentation assays. Significant differences were also calculated for scratched cultures of keratinocytes. Values of *p*<0.05 were considered significant.

## Results

### DCE and CS decrease intracellular GSH levels in human keratinocytes

**Figure 1:**
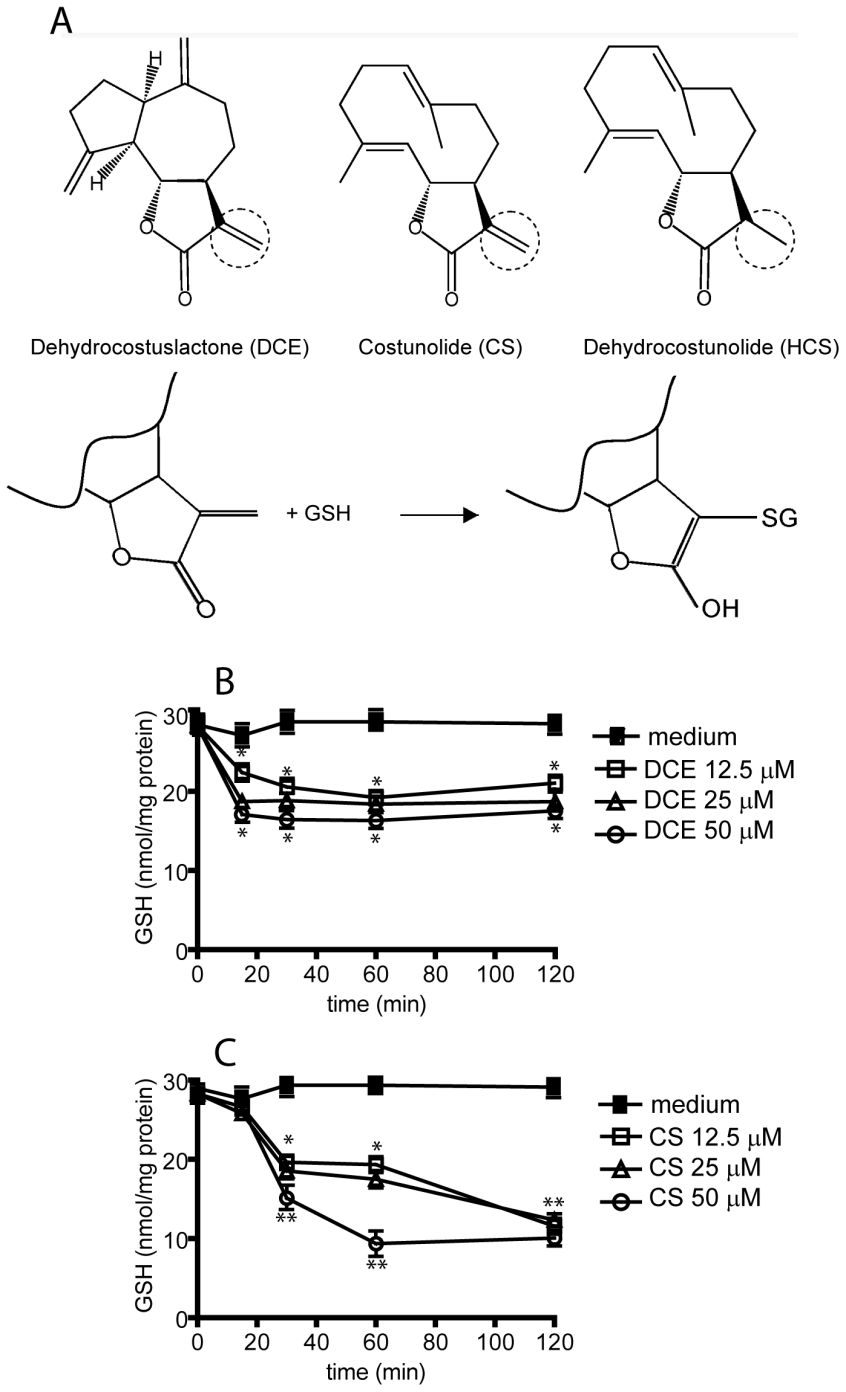
DCE and CS decrease intracellular GSH level in human keratinocytes. (A) Reaction scheme of GSH and CS or DCE interaction. DCE and CS, as well as other natural sesquiterpenes, have in common a reactive α-β-unsaturated carbonyl group that may react by a Michael-type addition with nucleophiles, such as cysteine sulfhydryl groups. DCE (B) and CS (C) time- and dose-dependently induce the drop in cellular GSH content measured by spectrophotometric analysis. Treatment of cultures with culture medium alone do not vary GSH release. Data are presented as means of GSH content ± SD of results of four independent experiments. Terpene-treated groups were compared to untreated groups (time 0). * *p*≤0.01 and ** *p*≤0.05.

DCE and CS determine a drop in levels of GSH in cells by functioning as potent Michael reaction acceptors (Figure 1A). DCE and CS can directly bind GSH and decrease its intracellular levels with a time kinetics and efficacy depending on cell types and the concentration used. Therefore, we firstly determined whether DCE and CS caused a significant GSH drop level also in human keratinocytes, and the appropriate doses and time of incubation of these two terpenes. As shown in Figure 1B and C, both DCE and CS induced dose- and time-dependently a substantial reduction in GSH concentration. GSH content in keratinocytes decreased more rapidly when treated with DCE compared to CS, although GSH drop after CS treatment was more prominent and long-lasting. Since DCE and CS determined similar GSH drop levels at the 12.5 µM dose and 1 h time-point after administration (Figure 1B and C), in the next series of experiments, these experimental conditions were chosen. These doses were not cytotoxic for keratinocytes, as assessed by measuring LDH release in the culture supernatants (data not shown).

### DCE and CS inhibit STAT3 and STAT1 whereas increase EGFR/ERK1/2 phosphorylation

**Figure 2:**
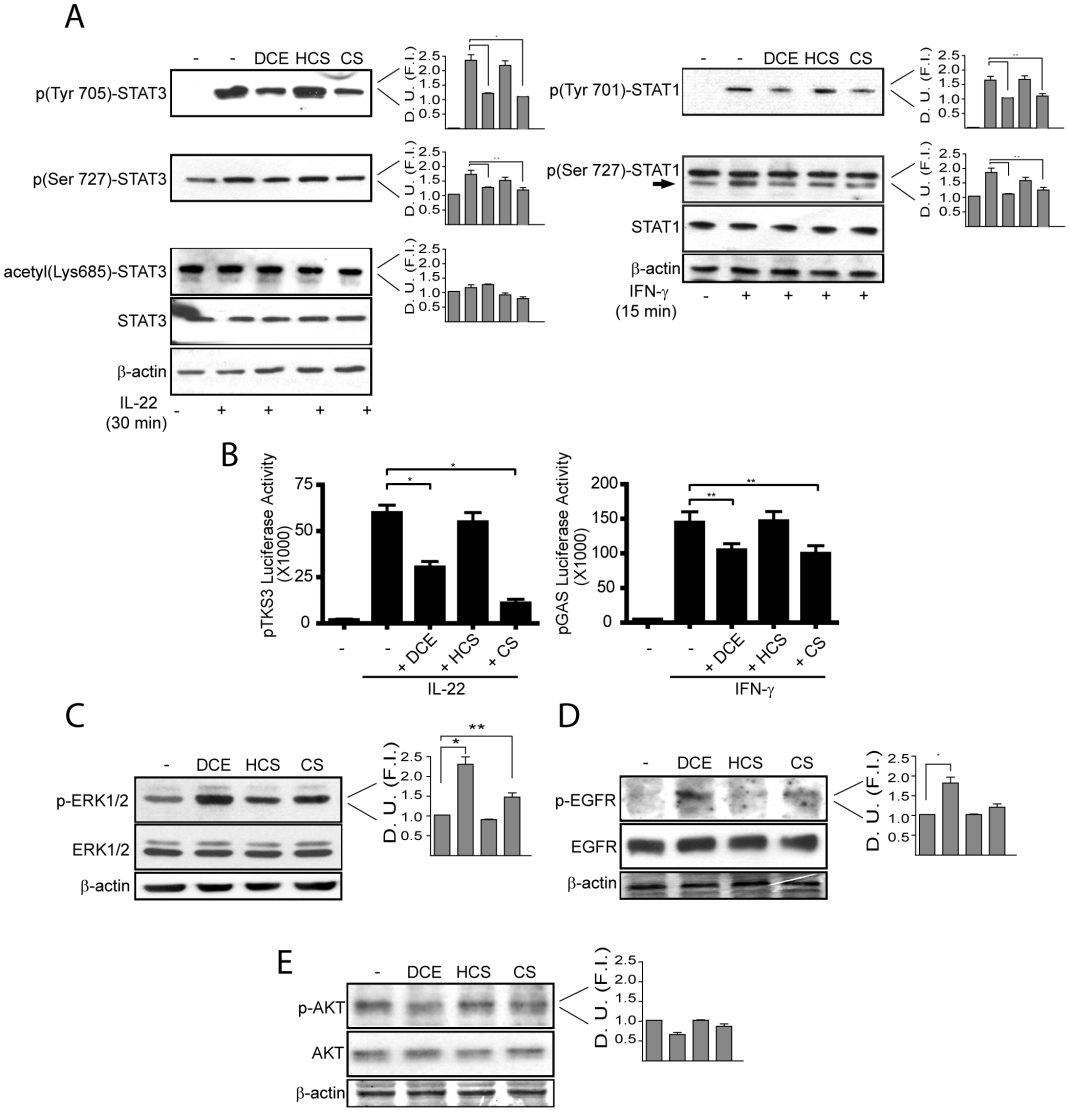
DCE and CS inhibit STAT3 and STAT1 signalings whereas increase EGFR/ERK1/2 phosphorylation. Total STAT3, Tyr705- or Ser727-phosphorylated STAT3, Lys685-acetylated STAT3, total STAT1, and Tyr701- or Ser727-phosphorylated STAT1 were detected by Western blotting of lysates from cultured human keratinocytes (A). STAT3 and STAT1 were analyzed in IL-22 and IFN-γ-activated keratinocyte cultures, respectively, treated or not with DCE, HCS or CS (all at 12.5 µM with 1-h pre-treatment). Graphs represent densitometric analyses of phospho-STAT3, acetylated STAT3, and phospho-STAT1 (D. U., Densitometric Units; F. I., Fold Induction). Effects of terpenes on the STAT3 or STAT1 activities were evaluated by assaying luciferase activity of STAT3 or STAT1 responsive plasmids (B). Data are expressed as means ± SD of *Firefly* luciferase values normalized to *Renilla* luciferase and µg of total proteins. * *p*≤0.01 and ** *p*≤0.05. Phospho-ERK1/2 (C), phospho-EGFR (D) and phospho-AKT (E) were evaluated by Western blotting on keratinocyte cultures treated with DCE, HCS or CS for 30 min. Graphs represent densitometric analyses. * *p*≤0.01 and ** *p*≤0.05. Immunoblot stainings are representative of 3 independent experiments performed on 3 different keratinocyte strains obtained from biopsies of different healthy donors.

Inflammatory and proliferative events in keratinocytes are crucially regulated by the transcription factors STAT3 and STAT1, which can be potently be induced by IL-22 and IFN-γ, respectively. In order to evaluate the effect of DCE and CS on STAT3 and STAT1 signalling pathway, Western blotting analysis and luciferase assay were performed using cultured human keratinocytes treated with IL-22 or IFN-γ, in presence or absence of DCE or CS. As negative control, we also pre-treated cells with dehydrocostonulide (HCS), the reduced form of CS. Critical step in STAT3 and STAT1 activation is their phosphorylation on specific tyrosine residues (Tyr705 for STAT3 and Tyr701 for STAT1) and serine residues (Ser727 for both STAT3 and STAT1). Moreover, STAT3 activation is proportional to acetylation in Lys685 residue, since this post-translational modification is required for phosphorylation in Tyr705. We found that DCE and CS potently decreased IL-22-induced Tyr705 phosphorylation of STAT3 without affecting the total amount of STAT3 protein (Figure 2A, left). Treatment with active terpenes also reverted the up-regulation of Ser727 phosphorylation of STAT3, which was constitutive in human keratinocytes. In contrast, Lys685 acetylation seemed to be not influenced by DCE and CS (Figure 2A, left). DCE or CS treatments, even though at lower level, inhibited IFN-γ -induced Tyr701 and Ser727 STAT1 phosphorylation (Figure 2A, right). Experiments of transient transfection with plasmids carrying luciferase gene and responsive to IL-22 or IFN-γ via STAT3 or STAT1, respectively, confirmed the results obtained by Western blotting, with STAT3 being more sensitive than STAT1 to DCE- or CS treatments (Figure 2B). As expected, HCS did not influence STAT3 or STAT1 activation and signalling (Figure 2B).

We next studied whether DCE and CS could influence other molecular pathways in human keratinocytes, and specifically, EGFR/ERK1/2 and AKT signalling, involved in cell survival/self-protection from inflammation and apoptosis, respectively. Interestingly, we found that both DCE and CS, but not HCS, significantly enhanced ERK1/2 and EGFR phosphorylation (Figure 2B and C). On the contrary, AKT activation did not significantly vary in terpene-stimulated keratinocytes compared to unstimulated or HCS-treated cells, although a slight decrease of AKT phosphorylation was observed especially in DCE-treated keratinocytes (Figure 2D; *p*>0.05).

### Inhibition of the IL-22- and IFN-γ-induced expression of inflammatory and regulatory genes by DCE and CS

**Figure 3:**
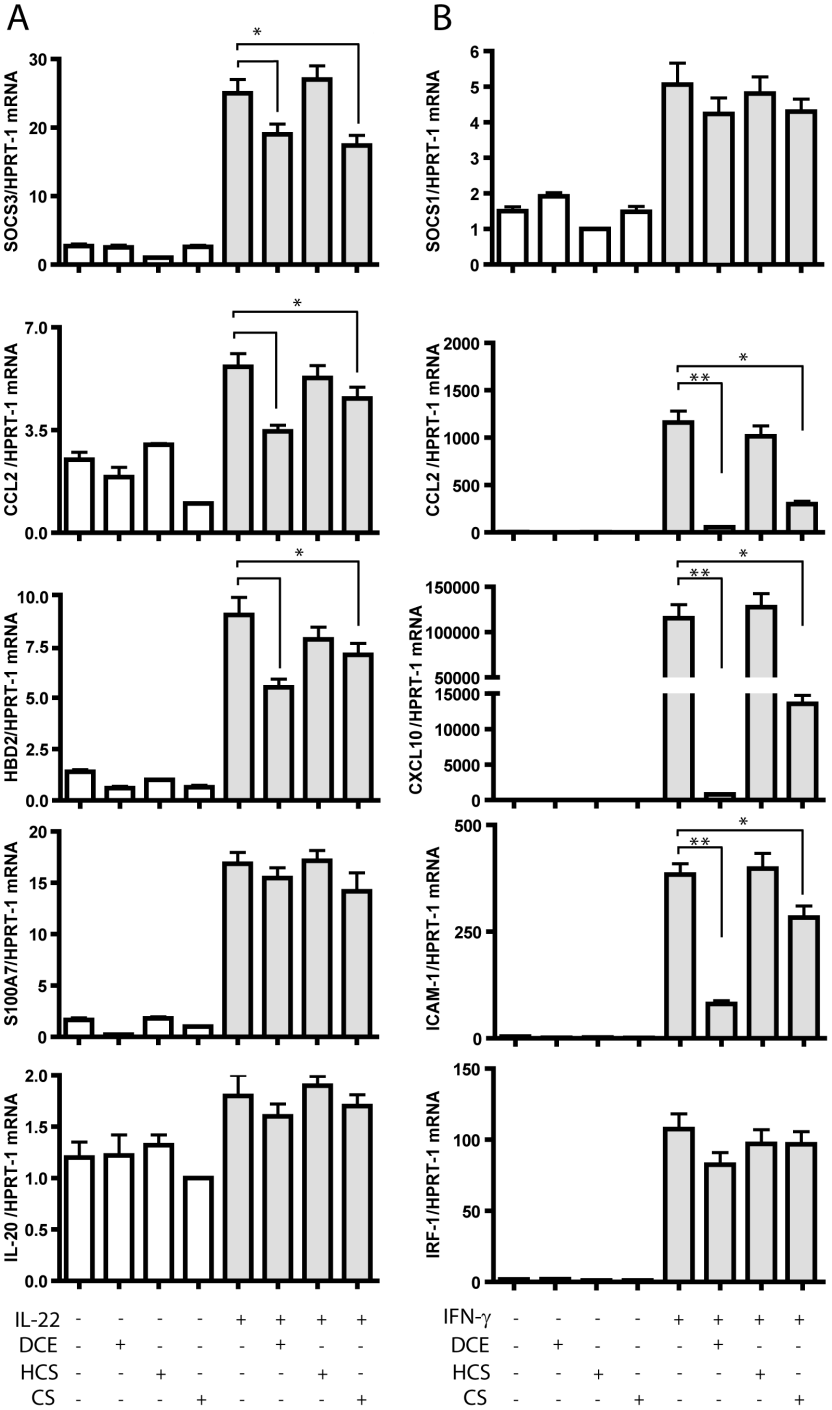
Modulation of the IL-22- and IFN-γ-induced gene expression by DCE and CS. The IL-22-induced SOCS3, CCL2, HBD-2, S100A7, IL-20 mRNA (A) and the IFN-γ-induced SOCS1, CCL2, CXCL10, ICAM-1, IRF-1 mRNA (B) expression were evaluated by real-time PCR analysis of RNA from cultured keratinocytes stimulated with the specific cytokines in presence or absence of DCE, HCS or CS, and normalized to HPRT-1 mRNA. * *p*≤0.01 and ** *p*≤0.05.

In the next series of experiments, we evaluated whether DCE and CS could influence the keratinocyte expression of genes induced by IL-22 or IFN-γ *via* STAT3 or STAT1, respectively. To this end, the expression of a variety of molecules involved in the induction or control of skin inflammation was studied by real-time PCR analysis in keratinocyte cultures pre-treated with DCE or CS and, then, stimulated with IL-22 or IFN-γ. We found that DCE and CS substantially reduced SOCS3, CCL2 and HBD-2 mRNA induced by IL-22 in human keratinocytes. In contrast, mRNA expression of S100A7 and IL-20 was not affected by terpene treatments (Figure 3A). Also CCL2, CXCL10 and ICAM-1 mRNA, potently induced by IFN-γ in keratinocytes, were inhibited by DCE and at lower extent by CS (Figure 3B). On the contrary, the expression of SOCS1 and IRF-1, a transcription factor controlled by STAT1 and in turn controlling genes sensitive to the IFN-γ/STAT1 pathway, were only slightly affected by terpene treatments (*p*>0.05, IFN-γ/DCE vs IFN-γ-treated sample) (Figure 3B). Importantly, DCE effect on CCL2, CXCL10 and ICAM-1 mRNA expression was extremely potent as it quite totally abrogated the expression of these inflammatory mediators (Figure 3B).

### DCE and CS inhibit keratinocyte proliferation by reducing the expression of genes involved in cell-cycle progression and by promoting mitotic arrest

**Figure 4:**
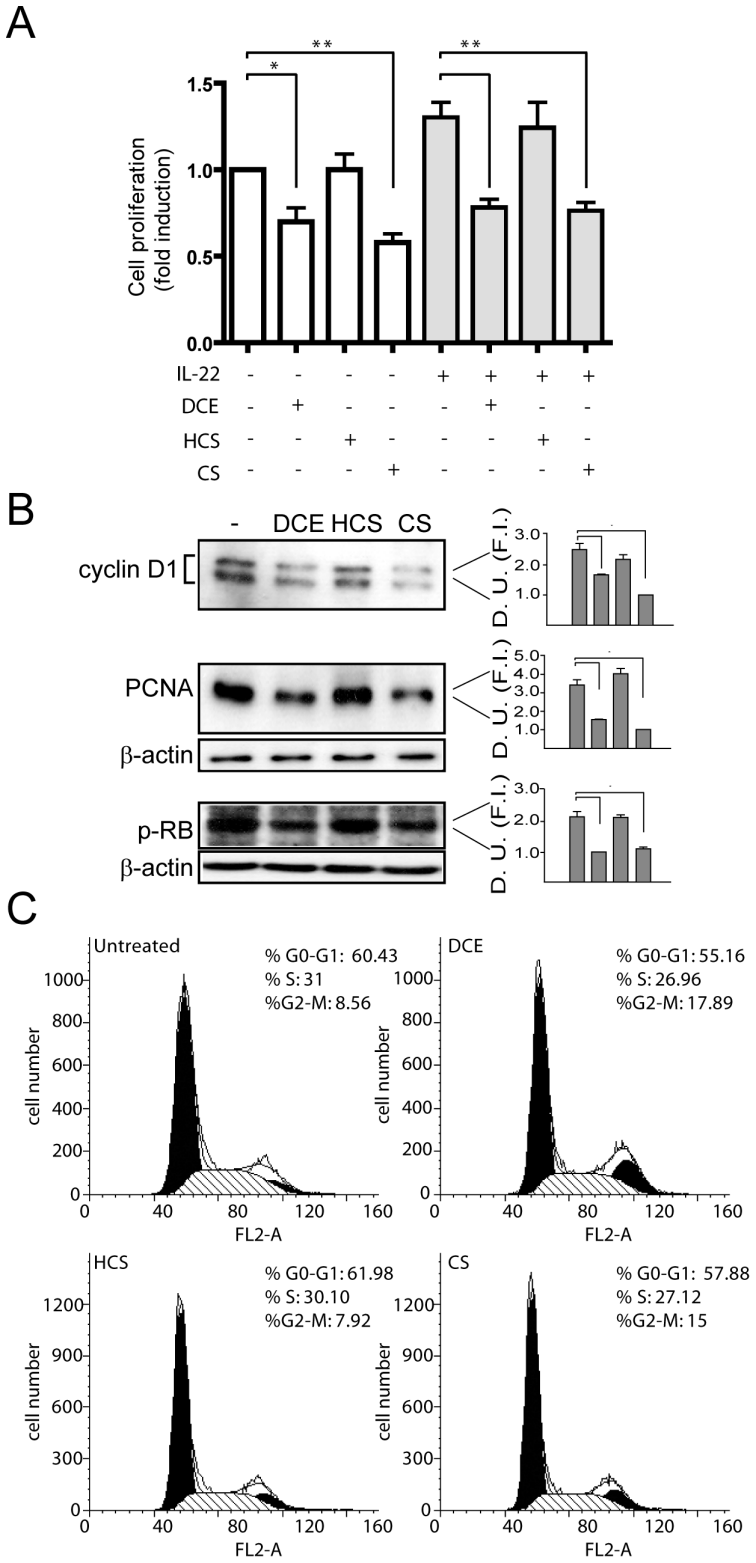
Inhibition of proliferation and cell-cycle progression of keratinocytes by DCE and CS. Proliferation of keratinocytes treated with DCE, CS or HCS either in presence or absence of IL-22 was proportional to crystal violet incorporation (A), which was measured with an ELISA reader after 2 d of culture. Data are expressed as fold induction of treated *vs.* untreated samples, which were given a value of 1. * *p*≤0.01 and ** *p*≤0.05. B) PCNA, cyclin D1, and pRB were analyzed by Western blotting. Graphs show densitometric values of PCNA, cyclin D1, and pRB staining on blots (D. U., Densitometric Units; F. I., Fold Induction). Western blots are representative of 3 independent experiments performed on 3 different keratinocyte strains obtained from biopsies of different healthy donors. * *p*≤0.01. C) Cell-cycle distribution analysis of cultured keratinocytes treated or not with DCE, CS, or HCS, for 16 h. The percentage of keratinocytes in G0-G1, S, and G2/M phases are indicated in each histograms.

To assess whether DCE and CS regulated keratinocyte growth and proliferation, we treated keratinocyte cultures with these active terpenes or HCS, in presence or absence of IL-22, a cytokine that regulates proliferation and differentiation processes in keratinocytes. Crystal violet assays demonstrated that DCE or CS, but not HCS, substantially reduced keratinocyte proliferation both in basal condition and after IL-22 treatment (Figure 4A). Importantly, DCE and CS treatments were efficacious in reducing the nuclear accumulation of cyclin D1, PCNA e p-RB, all molecules involved in cell-cycle progression and proliferation of keratinocytes (Figure 4B). Moreover, DCE and at lower extent CS, but not HCS, induced a cell-cycle arrest of keratinocytes in G2/M phases, and, in parallel, significantly decreased the percentage of cells in S and G0/G1 phases (Figure 4C).

### Apoptosis and migration are enhanced in keratinocytes treated with DCE and CS (Figure 5 and 6)

**Figure 5:**
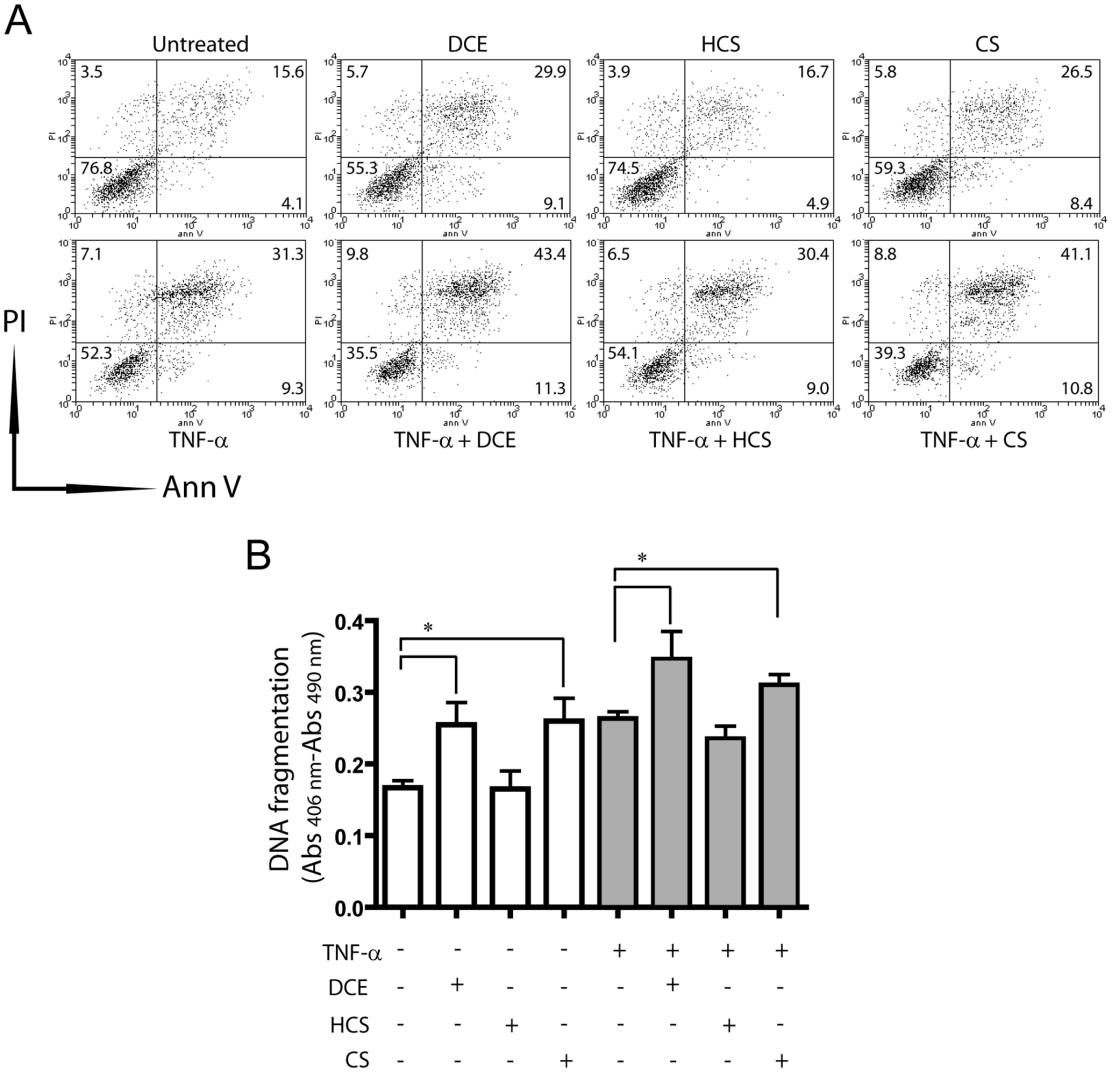
Apoptosis is enhanced in DCE- or CS-treated keratinocytes. Apoptosis of cultured keratinocytes treated with DCE, CS, or HCS in presence or absence of the pro-apoptotic stimulus TNF-α (50 ng/ml for 48 h) was examined by measuring Annexin/PI fluorescence through FACS analysis (A) or DNA fragmentation by ELISA (B). In A), a representative experiment of four performed is shown, with numbers indicating the percentage of PI^+^ (upper left), Ann V^+^ (lower right), PI/Ann V^+^ (upper right), or negative (lower left) cells. In B), * *p*≤0.05.

Sesquiterpene lactones have anti-apoptotic activity in various cancer cell lines, and function *via* GSH depletion and inhibition of STAT3 activation. To evaluate whether DCE and CS can influence apoptosis in keratinocytes, we treated keratinocyte cultures with active terpenes, administered alone or combined to the pro-apoptotic stimulus TNF-α, and quantified cell death by measuring annexin V/PI staining and DNA fragmentation levels. As shown in Figure 5A, both DCE and CS, but not HCS, significantly enhanced necrotic (PI^+^ cells), early apoptotic (Annexin V^+^) and late apoptotic (PI^+^/Annexin V^+^) keratinocytes. When DCE or CS and TNF-α were co-administered, viable keratinocytes further reduced (Figure 5A). In parallel, DNA fragmentation significantly enhanced in cells treated with DCE or CS, and even more in presence of DCE or CS used together with TNF-α (Figure 5B).

**Figure 6:**
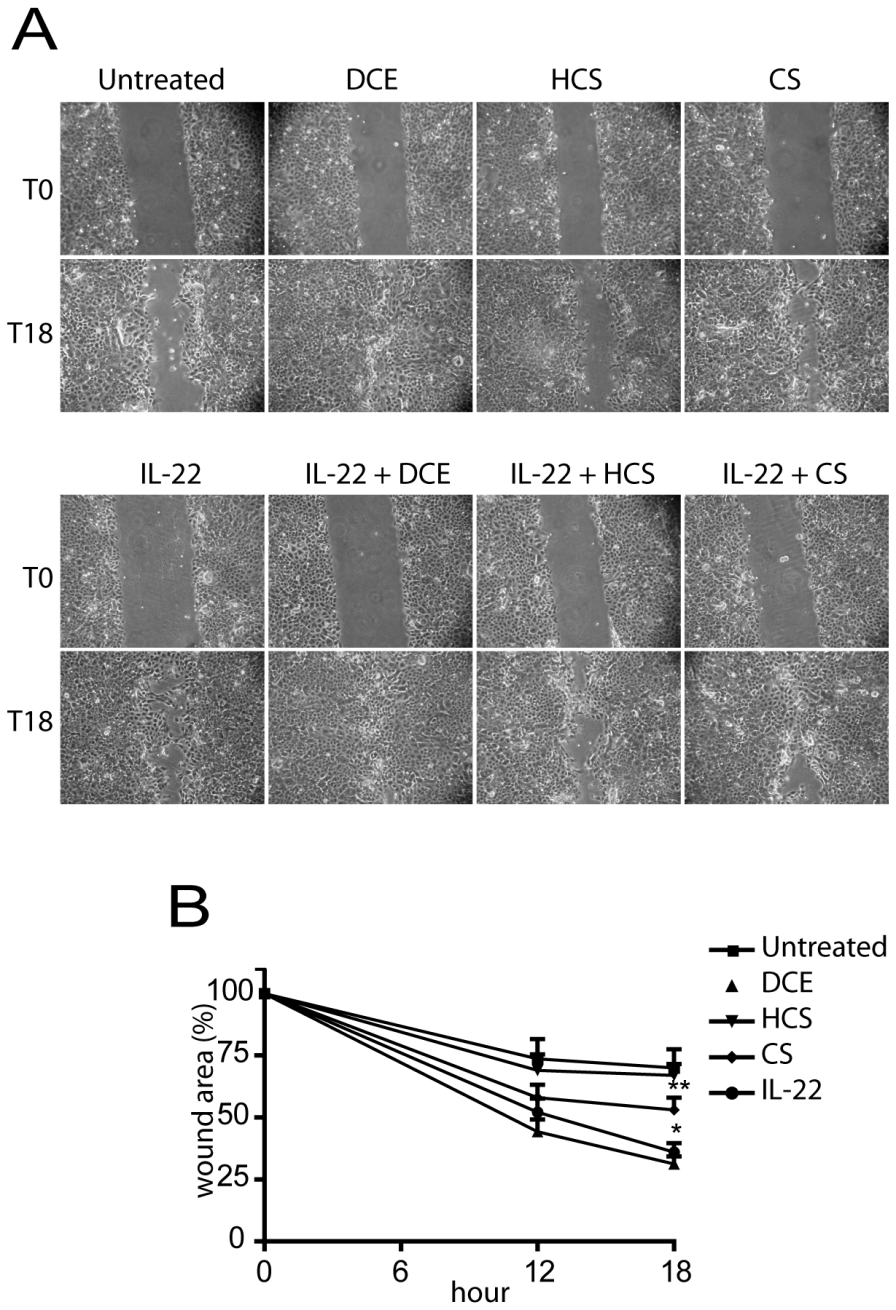
DCE and CS accelerates *in vitro* wound healing by enhancing keratinocyte migration. Scratch assays were carried out on scratched keratinocyte cultures incubated or not with 30 ng/ml IL-22 for 18 h, in presence of DCE, CS or HCS (A). Cultures were also treated with 10 µM mitomycin and stimulated as indicated in B). Residual gap between migrating keratinocytes is expressed as percentage of initial scratched area. * *p*≤0.01 and ** *p*≤0.05 vs untreated samples.

We finally studied the effects of DCE and CS on proliferative and migratory responses of keratinocytes to the cytokine IL-22, typically inducing regenerative programs in keratinocytes and involved in skin repair [7], [8], in functional *in vitro* scratch assay. Unexpectedly, CS and more efficiently DCE, but not HCS, enhanced IL-22-induced keratinocyte wound closure 18 h after scratching (Fig. 6*A*). CS and DCE significantly accelerated closure also of untreated scratched cultures, with an efficacy similar to IL-22. To understand whether DCE and CS effects on wound closure were due to a their capability to promote keratinocyte migration, cell monolayers used in scratch assays were pre-treated with mitomycin at concentration blocking proliferation, and then stimulated with DCE, CS or IL-22. As shown in the graph in Figure 6B, despite mitomycin treatment, keratinocyte cultures efficiently continued to close wounds *in vitro* when treated with DCE or CS, but not with HCS, indicating that these sesquiterpene lactones act on wound healing mainly by inducing keratinocyte migration.

## Discussion

In recent years, a number of effective treatments used for the therapy of psoriasis and based on the increase of local oxidative stress has become attractive [15], [16], [25], [26], [27]. These pro-oxidant treatments activate anti-proliferative and pro-apoptotic pathways in hyperproliferating keratinocytes, thus, counteracting the effects of inflammatory cytokines locally released by infiltrating leukocytes. With the aim at identifying new therapeutic molecules for skin disorders characterized by epidermal hyperproliferation, inflammation and marked resistance to apoptosis, in particular psoriasis, we examined the effects of two plant-derived pro-oxidant molecules, the sesquiterpene lactones DCE and CS, on regulating proliferative, immune, and apoptotic responses of human keratinocytes to inflammatory cytokines or in basal conditions. Previous studies demonstrated that the pro-oxidant effects of DCE and CS were correlated to their direct interaction with GSH that induces a rapid drop in intracellular GSH concentration. The oxidant stress can alter the function of protein through the reversible oxidation of the thiolic group of sensitive cysteine residues [28], [29]. In particular, these terpenes induce S-glutathionylation of STAT3 hindering its tyrosine phosphorylation and activation [22].

In this study, we showed that treatments of human keratinocytes with DCE and CS resulted in a substantial reduction of intracellular GSH levels and inhibition of STAT3 phosphorylation and activation. DCE and CS also inhibit STAT1 phosphorylation whereas significantly enhance EGFR and ERK1/2 cascades. These DCE- or CS-induced effects in keratinocytes are particularly important, since few molecules have been found so far to inhibit STAT3 and STAT1-dependent inflammatory pathways and, in parallel, to activate the anti-inflammatory EGFR and ERK1/2 molecules in keratinocytes. Other molecules influencing GSH levels, such as 1-buthionine sulphoximine (BSO), a glutamylcysteine synthase inhibitor, have been found to regulate STAT3 and ERK1/2 activation, even though they decrease synthesis of GSH rather than sequestering and oxidizing it. However, differently from DCE and CS, these molecules can have opposite effects depending on cell type and stimuli inducing STAT3 or ERK1/2. For instance, BSO inhibits STAT3 or ERK1/2 induced by leukemia inhibitory factor in cardiac myocytes, whereas it has no effects on IL-6-induced STAT3 activation in endothelial cells [30], [31]. On the other end, BSO can promote STAT3 activation in rat fibroblasts and epidermally derived A431 cells, as well as in rat livers [32].

The inhibition by DCE and CS of two primary inflammatory pathways in keratinocytes, the IL-22/STAT3 and IFN-γ/STAT1 signalings, resulted in a downregulation of the expression of genes involved in inflammatory processes. In particular, CCL2, CXCL10 and ICAM-1 mRNA, potently induced by IFN-γ in keratinocytes, and CCL2 and HBD-2 mRNA, induced by IL-22, were strongly inhibited by DCE and CS. The effects of terpene lactones were selective since not all the genes analyzed were significantly influenced by treatments. The most sensitive genes to DCE and CS action were those transcriptionally regulated by STAT3 (SOCS3, CCL2), and whose downregulation by mRNA degradation is driven by ERK1/2 (CXCL10, CCL2, ICAM-1) [8], [10]. Genes transcriptionally dependent by STAT1, such as SOCS1 and IRF-1 were only modestly influenced by DCE and CS, reflecting the weaker effect of terpene lactones on STAT1 as compared to STAT3 activation. This could depend on the capability of DCE and CS to induce rapid S-glutathionylation of STAT3, with concomitant decrease in STAT3 tyrosine phosphorylation, and not of STAT1. The inhibitory effect on STAT1 could be instead indirect, since DCE and CS are capable to inhibit phosphorylation of JAK tyrosine kinases upstream to STAT1 (i. e. JAK1 and JAK2). Further studies are required to test this hypothesis. On the other hand, we could not confirm previous findings showing that the inhibitory effect of DCE on constitutive STAT3 activation is mediated by an increase of SOCS expression [33].

A number of previous studies showed that DCE and CS inhibit proliferation and enhance apoptosis in different human cancer cells [34], [35], [36]. Specifically they can promote mitotic arrest accompanied by modulation of proteins involved in cell-cycle progression, including Chk2, Cdc25c, Cdk1, cyclin B1 [37]. We found that DCE or CS treatments efficiently induced apoptosis as well as inhibited proliferation in cultured human keratinocytes, either in basal conditions or in presence of IL-22. Proliferation inhibition was not due to cytotoxic effects of terpenes lactones but it was associated instead to cell-cycle arrest of keratinocytes in G2/M phases, and concomitant decreased expression of cyclin D1, PCNA and p-RB proteins. As a direct consequence of this cytostatic effect, keratinocytes treated with DCE or CS acquired cell motility and were more prone to migrate in functional *in vitro* scratch-wound assays in response to IL-22. CS and DCE significantly accelerated closure also of untreated scratched cultures, with an efficacy similar to IL-22, indicating that their effect is likely exerted on pathways constitutively activated in keratinocytes. At molecular level, this latter result can be related to the up-regulation of EGFR and ERK1/2, which notoriously are activated by mechanical injury and essential for keratinocyte migration during wound healing process [38]. Also downregulation of p-AKT could be involved in the enhancement of wound closure, as its inhibition is known to accelerate the scratch closure and potentiate the scratch-dependent stimulation of EGF-type growth factor genes [39].

In light of our findings, we can hypothesize the employment of DCE and CS in psoriasis might counteract the pro-inflammatory effects of IFN-γ and IL-22 on keratinocytes, as well as to inhibit hyperproliferation in the psoriatic epidermis. Moreover, DCE and CS might revert the apoptosis-resistant phenotype, which is due to intrinsic and acquired alterations of psoriatic keratinocytes. Among alterations, the abnormal activation of the SOCS1-3/PI3K/AKT and downstream anti-apoptotic NF-κB cascades play a pivotal role in rendering epidermis less susceptible to cytokine-induced apoptosis. While AKT seems to be only slightly downregulated by DCE or CS, NF-κB is strongly inhibited by serquiterpene from *Saussurea lappa* [21], and NF-κB inhibition by DCE is known to enhance TNF-α-induced apoptosis of cancer cells [20]. In addition, DCE- and CS-based treatments might be therapeutically relevant also for other skin diseases characterized by uncontrolled proliferation and deficient apoptosis in keratinocytes, including non-melanoma skin cancers. Further studies using terpene lactones *in vivo* in experimental models of skin hyperproliferative diseases are necessary to unveil their therapeutic efficacy, as recently demonstrated for other murine models of inflammatory diseases [40].
